# Micropolyps, Plasma Cells, and Pregnancy: Reevaluating Diagnostic and Therapeutic Strategies in Chronic Endometritis

**DOI:** 10.3390/jcm14186435

**Published:** 2025-09-12

**Authors:** Monika Szafarowska, Martyna Chirzyńska, Karolina Kurlenko, Magdalena Biela, Jacek Doniec, Krzysztof Łuszczyński, Aneta Ścieżyńska, Paweł Kamiński

**Affiliations:** 1 Department of Gynecology and Oncological Gynecology, Military Institute of Medicine National Research Institute, 128 Szaserów Street, 04-141 Warsaw, Poland; mszafarowska@wim.mil.pl (M.S.); mchirzynska@wim.mil.pl (M.C.); kkurlenko@wim.mil.pl (K.K.); mbiela@wim.mil.pl (M.B.); pkaminski@wim.mil.pl (P.K.); 2 Robotic Surgery Center, Military Institute of Medicine National Research Institute, 128 Szaserów Street, 04-141 Warsaw, Poland; jdoniec@wim.mil.pl; 3 Laboratory of Molecular Oncology and Innovative Therapies, Military Institute of Medicine National Research Institute, 128 Szaserów Street, 04-141 Warsaw, Poland; aneta.sciezynska@wum.edu.pl; 4 Department of Histology and Embryology, Medical University of Warsaw, 02-004 Warsaw, Poland

**Keywords:** infertility, diagnostic of chronic endometritis, micropolyps, endometrial biopsy, hysteroscopy, CD138

## Abstract

**Background/Objectives:** Chronic endometritis (CE) is a subclinical inflammation of the endometrium that affects female fertility. Although awareness of its impact on reproductive outcomes has increased significantly, clinical management—especially the diagnostic value of hysteroscopy and the effectiveness of perioperative antibiotic prophylaxis in improving fertility—remains unclear. **Methods:** This retrospective analysis involved 136 infertile women (30–44 years) who underwent diagnostic hysteroscopy between 2022 and 2023 at the Military Institute of Medicine in Warsaw. Women with intrauterine pathologies or other infertility factors were excluded. Hysteroscopic indicators of chronic endometritis (CE) included micropolyps and endometrial hyperemia. Endometrial biopsies were stained with CD138 and CE was diagnosed based on ≥5 plasma cells per 10 high-power fields. A single oral dose of azithromycin was administered post-procedure and pregnancy outcomes were assessed 12 months later. **Results:** CE was histologically confirmed in 29.2% of patients. The presence of micropolyps demonstrated a strong correlation with CE (*p* < 0.0001), although CE was also found in 21% of patients with normal hysteroscopic findings. While CE status did not significantly influence pregnancy rates, patients who received azithromycin exhibited a significantly higher conception rate (53% vs. 21%, *p* = 0.022). Additionally, secondary infertility was associated with higher reproductive success compared to primary infertility (54% vs. 24%, *p* = 0.022). **Conclusions:** Micropolyps are a specific hysteroscopic marker of CE. However, histologic inflammation markers may be present even in the absence of abnormal hysteroscopic findings. Furthermore, the routine use of antibiotic prophylaxis is associated with improved reproductive outcomes.

## 1. Introduction

Chronic endometritis (CE) is a benign condition characterized by persistent, low-grade inflammation of the endometrial lining, typically accompanied by subtle histological changes and minimal or absent clinical symptoms [1]. In spite of its often asymptomatic course, CE has attracted increasing attention due to its potential contribution to female reproductive dysfunction, particularly in the context of infertility, recurrent implantation failure (RIF), and recurrent pregnancy loss (RPL). The estimated prevalence of CE ranges from 0.8% to 19% in the general female population and may increase up to 56% among women suffering from infertility [2]. In contrast to acute endometritis, which presents with sudden and pronounced clinical symptoms, CE often develops slowly and may persist for extended periods, sometimes months or even years. The condition is frequently underdiagnosed due to its subtle and nonspecific clinical presentation, which may include pelvic pain, abnormal uterine bleeding, dyspareunia, and infertility [3].

**Figure 1:**
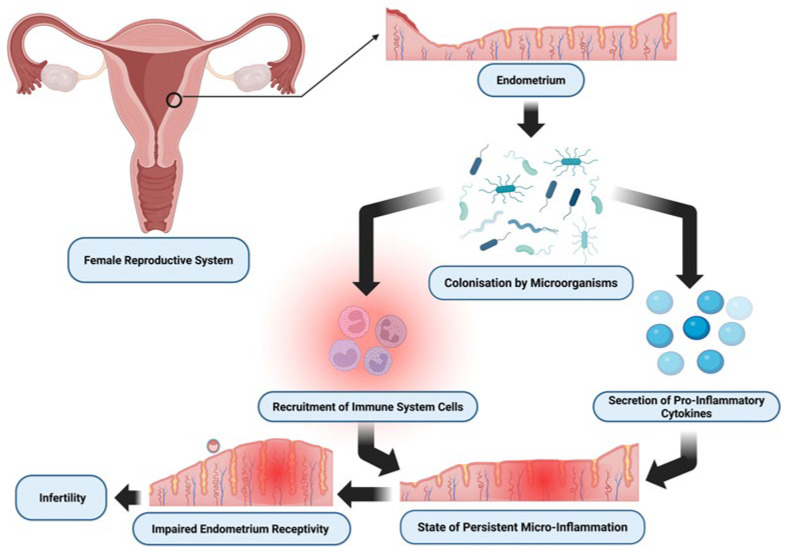
The molecular pathomechanism of chronic-endometritis-caused infertility. Created in BioRender. Łuszczyński, K. (2026).

The pathogenesis of CE is multifactorial, typically involving intrauterine colonization by microorganisms such as *Streptococcus* spp., *Staphylococcus* spp., *Escherichia coli*, *Enterococcus faecalis*, *Mycoplasma* spp., or *Ureaplasma* spp. [4,5]. These pathogens are believed to trigger an abnormal local immune response, characterized by the release of numerous pro-inflammatory cytokines, leading to impaired endometrial receptivity [6,7]. The morphological and functional alterations associated with CE include dysregulated cytokine signaling, infiltration of plasma cells, impaired decidualization, microvascular abnormalities, and disrupted uterine contractility [8]. Numerous studies have shown that the dysregulated inflammatory state of the endometrium in CE is associated with abnormal immune responses to bacterial components such as lipopolysaccharides, which can negatively affect implantation and result in infertility or miscarriage [8,9]. According to Buzzaccarini et al., impaired implantation in the context of CE may be associated with dysregulation of various cytokines, infiltration of leucocytes and plasma cells, altered decidualization, vascular abnormalities, and impaired uterine contractility (Figure 1).

Despite the clinical significance of CE, the diagnostic process remains complex and challenging. Histologic identification of plasma cell infiltration using CD138 immunohistochemical staining is considered the gold diagnostic standard; however, there is an ongoing scientific debate regarding the optimal threshold for the definitive diagnosis [10]. CD138 expression can also be detected in non-inflammatory uterine pathologies such as adenomyosis, endometrial polyps, and fibroids, which might impact interpretation and reduce specificity. In order to avoid overdiagnosis, it is important to recognize that CD138 can also be physiologically expressed in endometrial glandular epithelial cells and in numerous uterine pathologies [11,12].

In recent years, in accordance with the International Working Group for Standardization of Chronic Endometritis Diagnosis recommendations, hysteroscopy has been recognized as a valuable diagnostic tool, offering direct visualization of characteristic endometrial abnormalities associated with CE such as micropolyps, focal or diffuse hyperemia, hemorrhagic spots, and stromal edema [13]. Micropolyps appear as small (<1 mm) intrauterine growths with a distinct central core composed of connective tissue and small blood vessels, and are thought to result from localized inflammation [14]. Hyperemia manifests as small areas of increased vascularity or broader hyperemic regions interspersed with pale central spots (“strawberry-like” appearance) and may reflect inflammation-induced vasculopathy [15]. In a study by Furui et al. [16] authors stated that endometrial congestion was the only hysteroscopic feature significantly associated with chronic endometritis. However, the correlation between hysteroscopic findings and histologically confirmed CE varies among studies, and the clinical utility of these markers in predicting reproductive outcomes remains uncertain. In order to increase the predictive value of hysteroscopy and to reduce interobserver variability during the diagnostic process of chronic endometritis, Liu et al. created the scoring system, based on the hysteroscopic features such as diffuse hyperemia, focal hyperemia, hemorrhagic spots, dilated vessels, micropolyps, and polyps. Each finding was assigned an appropriate point value, and a cutoff score for the diagnosis of chronic endometritis was established at values greater than 2 points [17].

According to the literature, the gold standard for diagnosing chronic endometritis is hysteroscopic biopsy of the endometrium performed during the follicular phase of the menstrual cycle followed by histopathological analysis and confirmation of plasma cell infiltration [18,19]. It is important to emphasize that the histopathological diagnosis of chronic endometritis is not based on conventional morphological features such as increased stromal density, unsynchronized differentiation between endometrial epithelium, and stroma or superficial edema, but instead immunohistochemical staining for plasma cells is obligatory [20]. Immunohistochemistry staining for Syndecan-1 (CD-138), a specific marker of plasma cells, has significantly improved microscopic accuracy [21]. Nomiyama et al. described in a publication that in infertile patients with both endometrial polyps and infiltration of immune cells CE diagnosis was markedly higher (68.4%) in comparison to the group with negative CD138 staining (32.2%) or without endometrial polyps (28.3%) [22]. Nevertheless, the threshold number of plasma cells required for diagnosis remains uncertain [23]. A recent meta-analysis by Santoro et al. [10] suggested that ≥5 plasma cells in 10 high-power fields (HPF) is an appropriate criterion, with lower values requiring correlation with clinical and hysteroscopic findings.

Evidence suggests that CE may negatively impact reproductive outcomes by impairing endometrial receptivity [21]. CE has been reported in 2.8–56.8% of infertile patients, in 14–41% of those with RIF, and in 8–28% of those with RPL [2,24,25,26,27]. However, the literature shows discrepancies in diagnostic criteria and treatment approaches. The therapeutic benefits of antibiotic treatment for CE and the use of perioperative antibiotic prophylaxis after hysteroscopy remain unclear, particularly regarding their effect on pregnancy rates [28,29,30].

The aim of this study was to evaluate the diagnostic concordance between hysteroscopic findings and histopathological confirmation of CE in infertile women, and to assess the impact of CE and its treatment on subsequent pregnancy outcomes, with particular attention to the effect of perioperative antibiotic prophylaxis.

## 2. Materials and Methods

### 2.1. General Description of the Study

A retrospective study was conducted at the Department of Gynecology and Gynecological Oncology of the Military Institute of Medicine—National Research Institute in Warsaw. The study adhered to the principles outlined in the Declaration of Helsinki and received approval from the institutional Ethics Committee (approval no. 14/23). All participants were thoroughly informed about the study’s objectives, the intended use of their data, and the measures taken to ensure anonymity. Written informed consent was obtained from each participant. The research was supported by Statutory Grant No. 609 of the Military Institute of Medicine—National Research Institute, Warsaw, Poland.

### 2.2. The Patient Recruitment Process

A total of 136 infertile women aged 30 to 44 years, who underwent hysteroscopy at our clinic between 2022 and 2023 as part of their infertility evaluation, were enrolled in the study. All met the inclusion criteria and did not fulfill any of the exclusion criteria. The primary inclusion criteria were failed attempts to conceive for at least 12 months and an appropriate period of the menstrual cycle, defined as days 5 to 12 of the cycle. Exclusion criteria included uterine cavity diseases (endometrial polyps, submucosal myomas, intrauterine adhesions) or structural abnormalities (septate or didelphys uterus), as determined by transvaginal ultrasonography or hysteroscopy. Women who used oral contraception within three months of surgery or had known treatable reasons of reproductive failure were also excluded from the final analysis. No additional parameters, such as body weight, age, or comorbidities, were used to exclude participants from the research. Clinical data was gathered from medical records and responses to a standardized follow-up questionnaire.

### 2.3. Hysteroscopic Diagnosis and Sample Collection for Histological Analysis

In accordance with the standard infertility diagnostic protocol of our clinic, all patients underwent outpatient diagnostic minihysteroscopy. The procedures were performed using the vaginoscopic approach, as described by Prof. S. Bettocchi, without the use of analgesia or anesthesia [31]. All hysteroscopies were conducted during the follicular phase of the menstrual cycle, specifically between the 5th and 12th day. A rigid Office Continuous Flow Operative Hysteroscope (Karl Storz, Tuttlingen, Germany), with a 30-degree oblique view and an outer diameter of 4 or 5 mm, was used in all patients. Approximately 80% of the procedures were performed by a single experienced gynecologist (J.D.), ensuring a high level of consistency in both technique and interpretation. The remaining procedures were performed by M.S., M.B., M.C., K.K., and P.K.

During hysteroscopy, the cervical canal and uterine cavity were carefully assessed. The endometrium was evaluated for the presence of chronic endometritis in accordance with the International Working Group for Standardization of Chronic Endometritis Diagnosis. The presence of a strawberry-like aspect of the endometrial lining, focal hyperemia, hemorrhagic spots, micropolyposis, or stromal edema was considered characteristic of CE [5]. Targeted endometrial biopsies were obtained when abnormal findings were observed during hysteroscopy, while in cases of normal endometrial appearance, blind endometrial biopsies were performed. Following the procedure, all patients were advised to take oral antibiotic prophylaxis consisting of a single 1000 mg dose of azithromycin, which in the case of infertile women is a standard perioperative infection prevention procedure at the center performing the study, consistent with the literature recommendations for performing hysteroscopy in women with a history of pelvic inflammatory conditions [32].

Histological analysis assessed the presence of plasma cells using CD138 immunohistochemical staining. Chronic endometritis was diagnosed based on the identification of ≥5 CD138-positive plasma cells in 10 high-power fields (HPF) [10].

**Figure 2:**
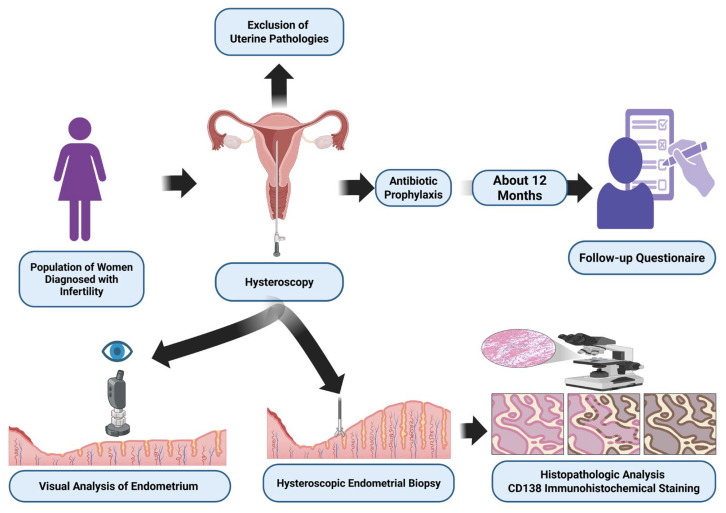
Study design for the assessment of diagnostic hysteroscopic features and reproductive outcomes in patients with CE. Created in BioRender. Łuszczyński, K. (2026).

On average, twelve months after the procedure, patients were asked to complete a follow-up questionnaire. The survey, developed and validated by the authors, included straightforward questions regarding the patients’ subjective experiences with outpatient hysteroscopy and their pregnancy outcomes. A graphical representation of the research methodology is shown in Figure 2.

### 2.4. Data Collection and Statistical Analysis

Collected data were analyzed using descriptive statistical methods. Categorical variables were presented as frequencies and percentages, while continuous variables were summarized using the mean, standard deviation (SD), and range (min-max) for normally distributed data, and the median, interquartile range (IQR), and range for non-normally distributed data. The Shapiro–Wilk test was employed to assess the normality of distributions [33]. Group comparisons for categorical variables were conducted using Fisher’s exact test [34] or the chi-squared test [35], depending on the expected frequencies within categories. For continuous variables, Student’s *t*-test [36] was applied to normally distributed data, and the Mann–Whitney U test [37] was used for data that did not meet the normality assumption. Univariable logistic regression analysis was performed to evaluate the effect of selected predictors on binary outcomes. Results were reported as odds ratios (OR) with corresponding 95% confidence intervals (CI) and *p*-values based on Wald’s test [38]. The predictive performance of binary predictors in relation to binary outcomes was assessed by calculating sensitivity, specificity, positive predictive value (PPV), and negative predictive value (NPV). All statistical tests were two-tailed, with the significance level set at α = 0.05. Analyses were conducted using R software, version 4.1.3 (R Core Team, 2022, Accessed on: 10 January 2025). The analyses relied on R’s built-in statistical computing capabilities and packages for data manipulation and modeling with script-based analysis enabled by R’s open-source architecture and package ecosystem [39].

Sensitivity, specificity, positive predictive value (PPV), and negative predictive value (NPV) were calculated from 2 × 2 contingency tables, comparing hysteroscopic findings with histopathological diagnosis of CE. Sensitivity was the proportion of histologically confirmed CE cases with positive hysteroscopic findings; specificity, the proportion of non-CE cases with negative hysteroscopic findings. PPV and NPV were defined accordingly.

Calculations followed the definitions and interpretation principles described by Glas et al. [40] and Stojanov et al. [41].

## 3. Results

### 3.1. Characteristics of the Study Population

Clinical and demographic characteristics of the study cohort, as well as the indications for hysteroscopic evaluation extracted from the medical records, are summarized in Table 1 and Table 2. Comorbid conditions were managed in collaboration with the patients’ primary physicians, ensuring the administration of appropriate therapeutic interventions throughout the study period.

### 3.2. Hysteroscopic Evaluation of the Study Population

During hysteroscopy performed in the cohort of infertile women participating in the study, a normal endometrial appearance was confirmed in 69% of women (*n* = 94). The most frequently identified feature highly suggestive of CE, based on hysteroscopic visualization, was the presence of micropolyps, observed in 21.3% of women (*n* = 29). Another finding was a hyperemic endometrium, present in 9.7% of cases (*n* = 13). No cases of stromal edema or hemorrhagic spots were identified during hysteroscopic assessment.

### 3.3. Histopathologic Analysis of the Acquired Endometrial Biopsy Samples

The histopathological analysis of endometrial biopsy specimens was normal in all patients. No cases of endometrial cancer, endometrial hyperplasia, polyps, or other histologic abnormalities were detected. As previously stated, the diagnosis of chronic endometritis was established when ≥5 plasma cells were identified in 10 HPFs; therefore, chronic endometritis, determined by immunohistochemical staining for CD138-positive plasma cells, was confirmed in 29.2% of women (*n* = 40).

### 3.4. Correlation of the Hysteroscopic Features with the Histopathologic Reports in the Diagnostic Process of Chronic Endometritis

The analysis revealed that, despite a normal endometrial appearance on hysteroscopic examination, histopathologically confirmed chronic endometritis was present in 21% of women (*n* = 20). Conversely, among women with hysteroscopic features highly suggestive of chronic endometritis, such as micropolyps and endometrial hyperemia (*n* = 42), histopathologic confirmation of the condition was obtained in only 47% of cases (*n* = 20). The strongest diagnostic correlation between hysteroscopic findings and histopathologic diagnosis of CE was observed in patients presenting with micropolyps (*p* < 0.0001). Interestingly, the occurrence of endometrial hyperemia—a finding commonly associated with chronic endometritis—did not differ significantly between the subgroups within the analyzed population of infertile women. This may be attributed to other underlying endometrial alterations unrelated to CE that could also cause hyperemia. The obtained results are presented in Table 3.

Following the initial data analysis, hysteroscopic markers were evaluated to determine the predictive value of hysteroscopic visualization in the diagnostic process of CE. Among women with histopathologically confirmed CE, hysteroscopic features characteristic of the condition were detected in 50% of patients during examination. Based on the collected data, the sensitivity, specificity, positive predictive value (PPV), and negative predictive value (NPV) of the hysteroscopic image of the endometrium in the diagnosis of CE were subsequently calculated. The results are presented in Table 4. The specificity of hysteroscopy in the diagnostic process of CE was 76.8%, and the negative predictive value was 78.8%.

### 3.5. The Comparison Between Hysteroscopic and Histopathologic Assessment of Endometrium During Chronic Endometritis Diagnosis as a Predictor of Pregnancy Success

On average, twelve months following the hysteroscopy, participants were asked to complete a questionnaire regarding their subjective experiences with the procedure as well as pregnancy outcomes. The survey was conducted among all patients; however, responses regarding pregnancy outcomes were received from only 60 women. Data analysis showed that pregnancy was achieved in 42% (*n* = 25) of cases analyzed. Fourteen of these were spontaneous pregnancies, and 11 were achieved after frozen embryo transfer. Subsequently, the association between pregnancy occurrence and hysteroscopic findings, as well as histopathological results, was analyzed. Among women with a normal endometrial appearance on hysteroscopy (*n* = 42), 45% became pregnant (*n* = 19). In contrast, in the group presenting hysteroscopic features highly suggestive of CE (*n* = 18), pregnancy was achieved in 33% of women (*n* = 6). However, these differences were not statistically significant. The results are presented in Table 5. Furthermore, no statistically significant correlation was observed between specific hysteroscopic features, such as micropolyps or endometrial hyperemia, and the pregnancy rate. The results are presented in Table 6.

Furthermore, no statistically significant correlation was observed between specific hysteroscopic features, such as micropolyps or endometrial hyperemia, and the pregnancy rate. The results are presented in Table 6.

In the next step of the analysis the endometrium histopathologic examination results were correlated with the pregnancy success. The analysis revealed that among women with histopathologically diagnosed chronic endometritis (*n* = 20), pregnancies were achieved in 45% of women (*n* = 9). In comparison, in the group in which chronic endometritis was excluded in the histopathological examination (*n* = 40), 40% of women became pregnant (*n* = 16). Therefore, no statistically significant correlation was observed between histopathologically confirmed CE and pregnancy rates. The results are presented in Table 7.

Among women with primary infertility (*n* = 25), 24% (*n* = 6) achieved pregnancy, while among those with secondary infertility (*n* = 35), 54% (*n* = 19) became pregnant. Logistic regression analysis showed a statistically significant association between secondary infertility and positive pregnancy outcome following diagnostic hysteroscopy with endometrial biopsy (*p* = 0.022). The results are presented in Table 8.

As intrauterine colonization by microorganisms is regarded as one of the main causes of chronic endometritis, we investigated whether perioperative azithromycin prophylaxis affected the pregnancy rate. Based on follow-up survey responses, 70.3% of women (*n* = 45) took the prescribed post-hysteroscopy antibiotic prophylaxis (Table 9). Among these women, 53% (*n* = 24) achieved pregnancy, compared with 21% (*n* = 4) in the group that did not take antibiotics (*n* = 19). Logistic regression analysis showed a statistically significant association between post-procedure antibiotic prophylaxis and positive pregnancy outcomes.

Complementary to the collection of clinical data, the follow-up survey assessed patient satisfaction with hysteroscopy. Based on the participants’ responses, 82.8% (*n* = 53) evaluated the outpatient hysteroscopy procedure performed without anesthesia or analgesia positively. The procedure was generally well accepted and tolerated. The median pain intensity reported during the procedure was 4.5 on the Visual Analogue Scale (VAS), with an interquartile range (IQR) of 2.0–6.0. The results are presented in Table 10.

## 4. Discussion

The presence of chronic endometritis (CE) in infertile women remains a diagnostic and therapeutic challenge. In our study, plasma cells detected by CD138 immunohistochemical staining were present in 29.2% of women, which is within the range reported in previous studies. Consistent with the findings of Cicinelli et al. [42], micropolyps were the most frequently observed hysteroscopic abnormality associated with CE in our cohort of infertile women (*p* < 0.0001). The diagnostic performance of micropolyps as a hysteroscopic marker for histopathologically confirmed CE in our study demonstrated a sensitivity of 50.0%, specificity of 76.8%, positive predictive value (PPV) of 47.1%, and negative predictive value (NPV) of 78.8%.

However, in contrast to the results reported by Cicinelli et al. [42], we found that even in the absence of hysteroscopic abnormalities, 21% of women had histopathologically confirmed CE. This observation supports earlier findings that other conditions associated with an impaired inflammatory state of the endometrium (IISE)—such as endometrial polyps, uterine myomas, adenomyosis, autoimmune disorders, diabetes, and oxidative stress—may also result in the presence of plasma cells in histopathological evaluations, complicating the differential diagnosis of CE [43]. Our results also differ partly from those of Song et al. [44], who, in a retrospective analysis of 1189 cases, reported endometrial hyperemia in 52.5% of patients, interstitial edema in 8.4%, and micropolyps in only 3.4%. In their study, the sensitivity, specificity, PPV, and NPV for hysteroscopic diagnosis of CE were 59.3%, 69.7%, 42.1%, and 82.8%, respectively. The authors further noted that the presence of more than one abnormality modestly increased diagnostic accuracy. Importantly, they emphasized that interobserver variability remains a major limitation of hysteroscopic assessment and that the absence of hysteroscopic features does not exclude the diagnosis of [45].

In our analysis, the overall diagnostic performance of hysteroscopy showed a sensitivity of 50.0% and specificity of 76.8%, with a PPV of 47.1% and NPV of 78.8%. The relatively high specificity and NPV indicate that a normal hysteroscopic image substantially reduces the likelihood of CE, suggesting a potential role for hysteroscopy in ruling out the condition in infertile patients. However, the moderate sensitivity implies that approximately half of histologically confirmed cases may be missed based solely on hysteroscopic appearance, limiting the value of hysteroscopy as a standalone diagnostic tool. As highlighted by Steinberg et al. [46], PPV and NPV are dependent on disease prevalence. Given that the prevalence of CE in our study was 29.2%, these values may differ in populations with higher or lower prevalence, which should be considered when applying our results to other clinical settings.

With respect to reproductive outcomes, we observed no statistically significant association between hysteroscopic features of CE and the likelihood of achieving pregnancy. However, analysis by infertility type revealed that women with secondary infertility had significantly higher pregnancy rates following diagnostic hysteroscopy compared with those with primary infertility, regardless of CE status. Specifically, pregnancy was achieved in 54% of women with secondary infertility versus 24% of those with primary infertility (*p* = 0.022). This difference may, at least in part, reflect the beneficial effect of endometrial scratching during hysteroscopic biopsy [21].

Finally, given that intrauterine colonization by microorganisms is considered one of the most important etiological factors in CE [18], we investigated the impact of perioperative azithromycin prophylaxis on pregnancy rates. Our analysis demonstrated a statistically significant benefit: 53% of women who received a single 1000 mg oral dose of azithromycin after hysteroscopy achieved pregnancy, compared with 21% in the non-treated group (*p* = 0.02). This finding suggests that targeted antibiotic prophylaxis may have a positive effect on reproductive outcomes in this patient population [18]. These findings are consistent with a work by Kitaya et al. [47], involving 421 patients with a history of recurrent implantation failure; the authors demonstrated that oral antibiotic therapy, specifically doxycycline as a first-line treatment and metronidazole combined with ciprofloxacin in doxycycline-resistant cases, was effective in treating chronic endometritis in 99.1% of patients. Moreover, the implemented treatment significantly improved reproductive outcomes, with a higher clinical pregnancy rate—45.7% vs. 34.1% and live birth rate—38.8% vs. 27.9%, in comparison to untreated women. Interestingly, authors also observed that chronic endometritis was more frequently diagnosed in couples with male factor infertility, suggesting a potential contributory role of the male partner in the pathogenesis or persistence of the disease. Furthermore, in a study performed by Cicinelli et al., researchers observed significantly higher pregnancy rate and live birth rate following IVF among women with successfully treated chronic endometritis compared to those with persistent CE after antibiotic therapy. They described that the pregnancy rate in the cured group was 65.2% in comparison to 33.0% in the persistent group (*p* = 0.039); and the live birth rate was 60.8% compared to 13.3%, respectively (*p* = 0.02) [2]. Moreover, authors suggested that antibiotic treatment promotes the normalization of endometrial abnormalities observed during hysteroscopy. Similarly, Cheng et al. reported a significantly higher clinical pregnancy rate and live birth rate among women with successfully treated CE in comparison to the group with persistent chronic endometritis [48].

Slightly different findings were reported by Qingyan Zhang et al., who analyzed their data to determine whether patients with antibiotic-cured chronic endometritis (CCE) had comparable pregnancy outcomes to those with non-chronic endometritis (NCE). The results revealed that the rate of early pregnancy loss was significantly higher in the CCE group, despite successful treatment, compared to the NCE group—21.2% vs. 14.2%. Based on these findings, authors proposed that in this group of women, the underlying cause of reproductive failure may be unrelated to chronic endometritis and instead caused by other factors [49]. Because of the clinical uncertainties regarding the effectiveness of chronic endometritis treatment, the medical community is awaiting the results of the prospective randomized clinical trial called “The effect of doxycycline on live birth rates in women with chronic endometritis suffering from recurrent miscarriage”. The outcomes of this study are believed to provide much needed explanations and help resolve existing controversies regarding the clinical impact of antibiotic therapy in the population of women suffering from chronic endometritis [50].

## 5. Conclusions

In our study, histopathological analysis confirmed chronic endometritis (CE) in nearly one-third of infertile women, despite a normal hysteroscopic appearance in a substantial proportion of cases. The strongest diagnostic correlation was observed with the presence of micropolyps during hysteroscopy; however, the sensitivity of hysteroscopic assessment alone was limited. These findings indicate the necessity of combining hysteroscopic evaluation with targeted endometrial biopsy and CD138 immunohistochemical staining to improve diagnostic accuracy.

Although no statistically significant association was found between CE and pregnancy rates, women with secondary infertility had significantly higher post-hysteroscopic pregnancy rates compared to those with primary infertility. Additionally, single-dose azithromycin prophylaxis following diagnostic hysteroscopy was associated with a statistically significant improvement in conception rates.

The study’s limitations include its retrospective design, single-center setting, limited follow-up period, and partial reliance on questionnaire-based data, which may introduce recall bias. Further prospective, controlled studies are needed to validate these results and to develop standardized diagnostic and therapeutic protocols for CE in infertility management.

## Figures and Tables

**Table 1:** Characteristics of the study population.

**Age [Years]**	
Median [IQR]	36.0 [33.0, 39.2]
Range	30–44
**BMI [kg/m^2^]**	
Median [IQR]	22.38 [20.36, 25.62]
Range	17.4–37.4
**Comorbidities**	**Number of Patients (Percentage)**
Hypothyroidism	31 (22.1%)
Insulin Resistance	24 (17.1%)
Endometriosis	14 (10%)
Hyperprolactinemia	13 (9.3%)
Polycystic Ovary Syndrome	7 (5%)
Other Disorders: Asthma, Hypertension, Migraine, Depression etc.	47 (34%)

**Table 2:** Medical indications for hysteroscopy.

**Characteristics of the Group**	**Number of Patients (Percentage)**
Total Number of Patients	136
**Indications for Hysteroscopy**	
Primary Infertility	67 (49.3%)
Secondary Infertility	69 (50.7%)

**Table 3:** The comparison of the results acquired from hysteroscopic and histopathologic analysis in the diagnostic process of CE.

**Chronic Endometritis Diagnosis in Histopathology**			
**Characteristics of the Group**		**Number of Patients (Percentage)**	
Total number of patients		136	
CD 138 < 5/10HPF		96 (69.8%)	
CD 138 ≥ 5/10HPF		40 (29.2%)	
**Endometrium in Hysteroscopy**			
**Characteristic**		**Number of Patients (Percentage)**	
Total Number of Patients		136	
Normal Endometrium		94 (69%)	
Micropolyps		29 (21.3%)	
Endometrial Hyperemia		13 (9.7%)	
Stromal Edema		0 (0%)	
Hemorrhagic Spots		0 (0%)	
**Correlation between Histopathology and Hysteroscopy**			
**Total Number of Patients**		136	
	**Total**	**CD 138 < 5/10HPF**	**CD 138 ≥ 5/10HPF**
Normal Endometrium	94	74	20
CE Suggestive Features	42	22	20
**Correlation between Histopathology and Hysteroscopic Features**			
**Endometrium Presence**	**CD 138 < 5/10HPF**	**CD 138 ≥ 5/10HPF**	***p*-value** **^1^**
Total	96	40	
Normal Endometrium	74 (77.0%)	20 (50.0%)	-
Micropolyps	11 (11.5%)	18 (45.0%)	<0.0001
Endometrial Hyperemia	11 (11.5%)	2 (5.0%)	NS
Stromal Edema	0 (0%)	0 (0%)	-
Hemorrhagic Spots	0 (0%)	0 (0%)	-

^1^ *p*-value ≤ 0.05 was considered statistically significant.

**Table 4:** The predictive value of the hysteroscopic image in the diagnostic process of CE.

**Parameter**	**Value [%]**
Sensitivity	50.0
Specificity	76.8
Positive Predictive Value	47.1
Negative Predictive Value	78.8

**Table 5:** Pregnancy outcomes following the procedure of outpatient hysteroscopy.

**Pregnancy Outcomes Following Hysteroscopy**					
**Characteristics**			**Number of Patients (Percentage)**		
Total Number of Patients			60		
Pregnant			25 (42%)		
Not Pregnant			35 (58%)		
**Correlation between Hysteroscopic Findings and Pregnancy Outcomes**					
**Endometrium Image in Hysteroscopy**	**Total**	**Pregnant**		**Not Pregnant**	***p*** **-value ^1^**
Normal Endometrium	42	19		23	-
CE Suggestive Features	18	6		12	NS

^1^ *p*-value ≤ 0.05 was considered statistically significant.

**Table 6:** The correlation between hysteroscopic markers of chronic endometritis and pregnancy rate.

**Endometrium Image in Hysteroscopy**	**OR ^1^**	**95%CI ^2^**	***p*** **-Value ^3^**
Normal Endometrium	-	-	
Micropolyps	0.692	0.161–2.65	0.598
Endometrial Hyperemia	0.484	0.064–2.53	0.416

^1^ OR = Odds Ratio, ^2^ CI = Confidence Interval, ^3^ *p*-value ≤ 0.05 was considered statistically significant.

**Table 7:** The correlation between histopathologically confirmed chronic endometritis and pregnancy outcomes.

**Correlation Between Histopathologic Findings and Pregnancy Outcomes**				
**Histopathologic Finding**	**Total**	**Pregnant**	**Not Pregnant**	***p*** **-Value ^1^**
Normal Endometrium	40	16	24	-
Diagnosed CE	20	9	11	NS

^1^ *p*-value ≤ 0.05 was considered statistically significant.

**Table 8:** The correlation between the type of infertility and pregnancy rate after hysteroscopy.

	**Primary Infertility**	**Secondary Infertility**	***p*** **-Value ^1^**
**Number of Patients**	25	35	
Positive Pregnancy Outcome	6 (24%)	19 (54%)	0.022
Negative Pregnancy Outcome	19 (76%)	16 (46%)	-

^1^ *p*-value ≤ 0.05 was considered statistically significant.

**Table 9:** The influence of post-hysteroscopy antibiotic prophylaxis on the likelihood of pregnancy.

**Characteristics of the Groups**				
**Total Number of Patients**		**64**		
	**With Prophylaxis**		**Without Prophylaxis**	
**Number of Patients**	45		19	
Positive Pregnancy Outcome	24 (53%)		4 (21%)	
Negative Pregnancy Outcome	21 (47%)		15 (79%)	
**Antibiotic Prophylaxis Influence on Pregnancy Likelihood**				
**Antibiotic Prophylaxis**	**OR ^1^**		**95%CI ^2^**	***p*-value ^3^**
Without Prophylaxis	-		-	
With Prophylaxis	4.29		1.32–16.9	0.022

^1^ OR = Odds Ratio, ^2^ CI = Confidence Interval, ^3^ *p*-value ≤ 0.05 was considered statistically significant.

**Table 10:** The patients’ satisfaction following the procedure of outpatient hysteroscopy.

**Patient Satisfaction Following Hysteroscopy**	
**Group Characteristic**	**Number of Patients**
Total Number of Patients	64
Positive Procedure Evaluation	53 (82.8%)
Negative Procedure Evaluation	11 (17.2%)
**Pain Intensity Reported During the Procedure (VAS)**	
Median	4.5
IQR ^1^	[2.0–6.0]

^1^ Interquartile Range.

## Data Availability

All relevant data are included within the manuscript. The raw data are available on request from the main author.

## Abbreviations

The following abbreviations are used in this manuscript:

CE	Chronic Endometritis
RIF	Recurrent Implantation Failure
RPL	Recurrent Pregnancy Loss
HPF	High Power Fields
SD	Standard Deviation
IQR	Interquartile Range
CI	Confidence Intervals
PPV	Positive Predictive Value
NPV	Negative Predictive Value
IISE	Inflammatory State of the Endometrium
CCE	Cured Chronic Endometritis
NCE	Non-Chronic Endometritis
VAS	Visual Analogue Scale
